# An update on lateral flow immunoassay for the rapid detection of SARS-CoV-2 antibodies

**DOI:** 10.3934/microbiol.2023020

**Published:** 2023-04-13

**Authors:** Lucia Spicuzza, Davide Campagna, Chiara Di Maria, Enrico Sciacca, Salvatore Mancuso, Carlo Vancheri, Gianluca Sambataro

**Affiliations:** Department of Clinical and Experimental Medicine, University of Catania, Catania, Italy

**Keywords:** lateral flow immunoassay, COVID-19, SARS-CoV-2, IgG, IgM, rapid antibody test, point of care test, neutralizing antibodies

## Abstract

Over the last three years, after the outbreak of the COVID-19 pandemic, an unprecedented number of novel diagnostic tests have been developed. Assays to evaluate the immune response to SARS-CoV-2 have been widely considered as part of the control strategy. The lateral flow immunoassay (LFIA), to detect both IgM and IgG against SARS-CoV-2, has been widely studied as a point-of-care (POC) test. Compared to laboratory tests, LFIAs are faster, cheaper and user-friendly, thus available also in areas with low economic resources. Soon after the onset of the pandemic, numerous kits for rapid antibody detection were put on the market with an emergency use authorization. However, since then, scientists have tried to better define the accuracy of these tests and their usefulness in different contexts. In fact, while during the first phase of the pandemic LFIAs for antibody detection were auxiliary to molecular tests for the diagnosis of COVID-19, successively these tests became a tool of seroprevalence surveillance to address infection control policies. When in 2021 a massive vaccination campaign was implemented worldwide, the interest in LFIA reemerged due to the need to establish the extent and the longevity of immunization in the vaccinated population and to establish priorities to guide health policies in low-income countries with limited access to vaccines. Here, we summarize the accuracy, the advantages and limits of LFIAs as POC tests for antibody detection, highlighting the efforts that have been made to improve this technology over the last few years.

## 1. Introduction

Analysis of biomarkers is a fundamental aspect of medical evaluation and point-of-care (POC) tests have become important tools when a rapid and sensitive analysis is required outside the laboratory. Lateral flow assay is one of the most important and widely used biosensor platform for POC diagnostic [1]. Soon after the onset of the coronavirus disease 2019 (COVID-19) pandemic in December 2019, a large number of serological tests and methodologies have been developed within a short period of time to characterize the immune response to SARS-CoV-2 [2].

After three years from its initiation, the pandemic, although significantly slowed, is far to be eradicated. The current scenario includes wide spread of vaccination in high- and middle-income countries, where >80% of the population has received at least one dose, low rates of vaccination in low-income countries and the emergence of a number of new virus variants [3]. In this context, serology is still important in fighting the pandemic and in the development of control strategies. Serological tests can be now deployed as surveillance tools to understand the epidemiological risk, particularly in those countries where vaccination rates are low and to monitor the longevity of the immune response induced by natural infection or vaccination [4]–[6]. While serological assays may provide a helpful tool to understand the extent of immunity across various communities and countries, assays performed in laboratories are generally expensive and time-consuming, thus difficult to be employed on large scale surveys.

Lateral flow immunoassay (LFIA) for qualitative antibody detection, is performed outside the laboratory and provides results within minutes [7]. For the detection of anti-SARS-CoV-2 antibodies these assays should theoretically fulfill the WHO criteria for POC tests being Affordable, Sensitive, Specific, User-friendly, Rapid and robust, Equipment-free and Deliverable (ASSURED criteria) [8].

These criteria have become increasingly important during the pandemic, particularly because of the significant health and economic impact and disparity at a global level [9]. In 2020 a large number of such assays where commercialized, with an emergency use authorization (EUA), and used as complementary tests to RT-PCR in suspected cases, when these were negative or in those settings where molecular testing was expensive or unavailable. Some of these assays showed acceptable accuracy while others were retired from the market. By the beginning of 2021 a large part of the population was immunized by natural exposure to the virus and successively a massive vaccination campaign was implemented worldwide. At this point, the interest in LFIA resurfaced due to the need to ascertain the extent of seroprevalence in the naturally immunized as well as in the vaccinated population, to establish the longevity of this immunization and priorities to guide public health policies. Aware of the need to use affordable POC tests for this purpose, companies continued to develop assays with improved performance implementing new materials and improving the immunochromatography technology.

In this brief review we will summarize the current knowledge on the use and development of the lateral-flow technology for anti-SARS-CoV-2 antibody detection, emphasizing lights and shadows of this rapidly growing bioanalytical tool.

## 2. Overview on serological detection

### 2.1. Antibodies production against SARS-CoV-2

In humans, SARS-CoV-2 infection triggers both innate and adaptive immune system responses [10]. In brief, after airway epithelium is infected with SARS-CoV-2 the virus can cause cell damage and lysis. The virus-infected epithelium presents the antigen to CD8 T cells which show cytotoxicity and induce apoptosis of the infected epithelial cells. Dendritic cells present the antigen to CD4 T cells inducing differentiation to Th1, Th17 and T follicular helper (TFH). The TFH cells help B lymphocytes, responsible for immunological memory, to develop into plasma cells and promote the production of IgM, IgA and IgG isotype specific antibodies [10].

Each of these antibodies reveals a distinct kinetic and seroconversion timeline in response to SARS-CoV-2 infection, also depending on the state of the host immune system [11],[12]. Patients with severe COVID-19 disease and high viral load exhibit higher antibody titers than patients with mild disease [12]. Studies on kinetic show that in the first phase after the infection antibodies are produced against the nucleocapsid protein (N) and successively against the spike component (S) [13].

The seroconversion for IgM occurs generally from 4 to14 days after symptoms onset, so after 8 days on average IgM can be easily measured in the serum, reaching a plateau after around 3–4 weeks [14],[15]. The increase in IgA titer occurs after 6–8 days up to 21 days from symptoms onset, with a higher concentration compared to IgG, but a lower specificity [16],[17].

Serum IgG levels remain low in the early stage of the disease and gradually increase after the first week, reaching a plateau after 20–25 days, persisting up to 8 weeks [12],[14],[18]. In one study, the median seroconversion time was 12 days for IgM and 14 days for IgG [19]. The IgG levels remain high for several months, and are still detectable after one year [20].

In one study, the IgG titer reached a peak after 30 days (mean 170 BAU/mL) from the infection with SARS-CoV-2, and after a slow gradually decrease, the antibodies remained detectable (mean 50 BAU/mL) up to 300 days [21]. In an unvaccinated population with previous COVID-19, after 15 months from disease onset 80% of the patients were still IgG-positive [22]. Due to the implementation of the vaccination campaigns worldwide, data on the persistence of naturally produced antibodies for periods longer than one year are difficult to obtain.

However, some data are available from the immune response to SARS-CoV and Middle East Respiratory Syndrome (MERS)-CoV [23]. Although important differences have been found between MERS-CoV, SARS-CoV and SARS-CoV-2 in terms of receptor binding, immune cellular response and cytokines production, the antibody responses do not significantly differ [14],[23],[24]. A systematic review comparing studies on humoral response found that the median time to detection was similar across different antibodies for SARS-CoV (12 days; IQR 8–15.2 days) and SARS-CoV-2 (11 days; IQR 7.25–14 days), and slightly longer for MERS-CoV (16 days; IQR 13–19 days) [14]. A comparison of the antibody response over the time in the three diseases is shown in Table 1.

**Table 1.** Antibody detection over the time in SARS-CoV, MERS and SARS-CoV-2.

		Time of first detection	Antibody peak	Longevity of seroconversion
SARS-CoV	IgM	3–6 days	3–4 weeks	Undetectable after 6 months
	IgG	5–8 days	1–4 months	Persisting after 2 years in >90% of patients Persisting in ≈50% of patients after 3 years
MERS	IgM	2–12 days	3–4 weeks	NA
	IgG	2–12 days	3–4 weeks	Detectable 13 months after the infection
SARS-CoV-2	IgM	4–14 days	2–3 weeks	Mainly undetectable after 3 months
	IgG	7–18 days	3–4 weeks	After 15 months detectable in 80% of unvaccinated patients

Time intervals are those reported by different studies; NA: not available

### 2.2. Serological assays

Assays for the detection of antibodies against SARS-CoV-2 in human serum rely on the principle of antigen-antibody specific binding. These assays can provide information on the immune state of infected individuals in both a qualitative manner, assessing only the antibody isotype and in a quantitative manner, assessing both the antibody isotype and quantity (titer) [25].

In brief, the SARS-CoV-2 is constituted by the nucleocapsid protein (N), forming the capsid outside the genome and by a further envelope characterized by three structural proteins: membrane protein (M), spike protein (S), formed by the S1 and S2 subunits and envelope protein (E) [26]. Early observations revealed that SARS-CoV-2 infection in humans produces abundant antibodies against the N protein, showing cross-reactivity with other coronavirus and with the new variants of interest (VOC) [24],[26]. Antibodies against the S protein are more specific and the immunogenic characteristics of the S protein have been of great interest as this is a main target for neutralizing antibodies involved in vaccine response [27],[28]. The available serological assays can detect antibodies against both the N and the S protein (including the S1 and S2 subunits) and the receptor-binding domain (RBD) [28],[29]. The combination of N and S strengthens the accuracy of serodiagnostic performance [30].

The most commonly used quantitative tests include the Enzyme-Linked Immunosorbent Assay (ELISA) and the Chemiluminescent Immunoassay (CLIA). Among different methods to perform ELISA, the indirect ELISA is one of the most commonly used to detect antibodies against SARS-CoV-2. The principle of this assay is to coat virus proteins, N, S, S1, or RBD, on the solid phase carrier that binds with serum antibody and enzyme-linked antibody to produce a chromogenic reaction [25],[31]. Over the last years the ELISA technology has become more sophisticated so that new methods have been developed able to detect antibodies independently of the SARS-CoV-2 variant [32]. The CLIA method is an assay that combines the chemiluminescence technique with immunochemical reactions. CLIA utilizes chemical probes generating light emission through a chemical reaction to label the antibody and has been pointed as one of the most accurate methods to detect antibody against SARS-CoV-2 [31]. Recently developed multiplex immunoassays are able to simultaneously detect antibodies against N, S and RBD antigens providing a greater accuracy [33].

After the onset of COVID-19 pandemic a large number of LFIAs became available as the US Food and Drug Administration (FDA) through the EUA committee has accelerated market accessibility. Although in an early phase of the pandemic these assays were used in parallel with direct tests for virus detection to improve diagnosis, successively, these assays have been widely used for seroprevalence studies in high risk groups and for a better comprehension of the SARS-CoV-2 immune response, thus improving our understanding of correlates of protection in humans [34],[35]. There is little doubt that studies on serology have significantly contributed to build the intricate road leading to the development of vaccines against SARS-CoV-2 and to the difficult task to estimate induced immunity on large scale and in single cases [36].

Although quantitative tests are highly sensitive and specific, they require time to obtain results, usually from 2 to 8 hours, and multiple resources since they must be performed in laboratories [31]. Qualitative POC tests, based on lateral flow technology, provide a valuable alternative to centralized assays being easy to use, fast, portable, stable and cost-effective [37].

### 2.3. Principles of lateral flow technology

The lateral flow assay is an immunochemical method of analysis based on the principle of thin layer chromatography carried out using special test strips [38]. This method was first introduced in1984 as an easy-to-use urine-based pregnancy test for domestic application [39].

**Figure 1.**
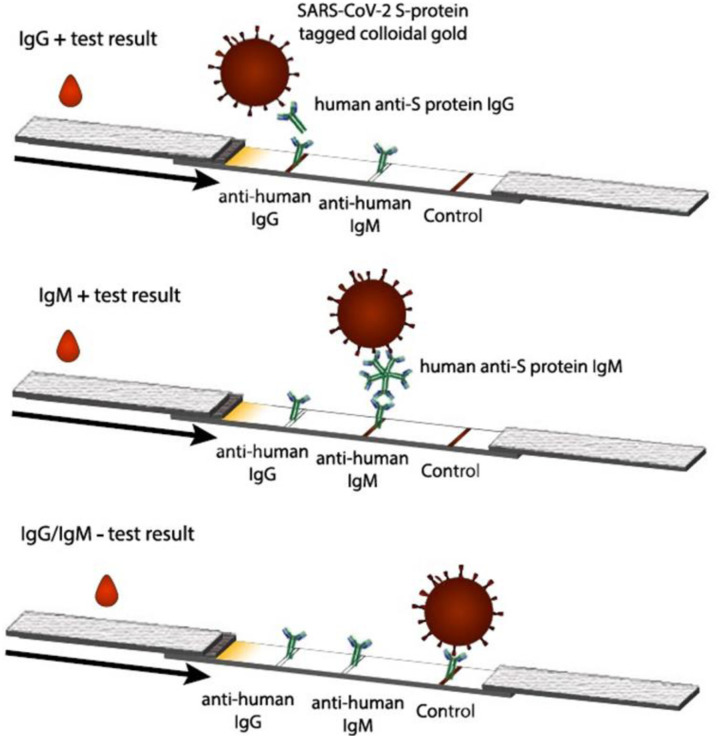
Schematic view of a LFIA for the detection of SARS-CoV-2 IgG/IgM. If antibodies are present in the sample a complex is formed with the SARS-CoV-2 antigen tagged nanoparticles and the anti-human IgG immobilized on the nitrocellulose membrane. A signal will be observed in the test line where the anti-IgG is captured, a signal must be visible also on the control line indicating the correct execution of the test (from Ernst et al. ref n.40).

Since then, this technology has continuously advanced with a great amount of commercial research leading to new clinical applications, new processing methods and improvements in reporting species and reading results [40]. A typical LFIA kit consists of: 1) a sample pad 2) a conjugate release pad 3) a nitrocellulose membrane containing immobilized antibodies on its surface 4) an adsorbent pad 5) a plastic backing 6) biological reagents [40]. (Figure 1). Typically, each test cartridge has two detection bands (test lines) and one distal band (control) that appears when the sample has flowed to the end of the strip. In brief, to perform the assay the liquid sample, usually microliters of finger-prick whole blood, is added to the sample pad and flows, driven by capillary force, to the conjugate pad. During this process the target in the sample is captured by specific antigen or antibody-coated nanoparticles present in the conjugate pad. The complex formed continues to flow to the nitrocellulose membrane binding the embedded specific antigen or antibody. Within minutes, if the target molecule is present in the sample, it is captured forming a specific signal in the test line. In the zone where a control line is present, unbound signaling materials or a second type of particles are captured. The signal in the control line indicates that a sufficient amount of fluid was applied for sufficient flow and release of particles, and the correct performance of the assay [41].

LFIA to detect antibodies against SARS-CoV-2 is based on the capture of specific IgG or IgM by immobilized anti-human IgG or IgM, respectively [40]–[42]. The capture occurs after the specific recognition of the antibody in the sample by SARS-CoV-2 antigen tagged nanoparticles [17],[40]. As in other serological assays, antigens consist of the S protein, the N protein or the RBD [17]. Commonly, the nanoparticles used for these tests, which are responsible for the signal intensity on the strip, consist of colloidal gold (AuNP) [43]. In fact, the small size of these particles (10–100 nm) allows significant accumulation at the test lines and their intense color helps the detection of low quantity of analytes [43].

### 2.4. Systems to improve sensitivity and accuracy of LFIA

After the onset of the pandemic, efforts have been done to speed up the production of more sensitive ad accurate LFIA kits, mainly enhancing the performance of existing technology. Key aspects include the improvement in: 1) sample collection/handling (e.g., concentration and preparation); 2) recognition and signal generation technologies; 3) readout and signal transduction technologies; 4) design of the cartridge [44].

Pre-concentration of the sample is feasible, but impractical for POC tests. Signal enhancement is a goal that can be pursued through the development of innovative optical reporter systems and the development of new nanotechnology-based approaches [7],[45]–[47]. As mentioned, in the field of nanoparticles, the first common approach has been to modify the size or structure of commonly used AuNP, which are both critical factors affecting the sensitivity of LFIA [7]. In addition, some other innovative labeling probes are currently under evaluation [38]–[40]. As an example, the technology using quantum dots, also known as fluorescent semiconductor nanocrystals, is under evaluation in the field of SARS-CoV-2 diagnostic [50]. Quantum dots are outstanding fluorescent materials with unique optical and electrical properties and many LFIAs have been produced using these labels with enhanced sensitivity [50],[51]. Other label materials are under evaluation, including latex particles, magnetic nanoparticles and more recently carbon, silica and europium nanoparticles [7],[47]. In some cases combined label materials have been used to improve sensitivity [52]. Innovations in the conjugation processes and protocols are also under evaluation to improve the analytical performance [7].

Although the production on large scale of assays using new technologies is challenging, mainly for the availability of new sophisticated label materials, for many of these results are encouraging, showing a significantly greater performance compared with gold nanoparticle-based commercial kits [47],[50],[53],[54]. Another method to improve sensitivity is the use of an external reader to amplify the signal [20],[46]. In fact, one problem with the interpretation of these tests is that the signal on the test line can vary in intensity so that a reading with naked eyes can be misleading and operator-dependent.

One system to improve the performance of these assays is the assessment of the signal with a spectrophotometer [55],[56]. Recently, Pieri and co-workers found that the combined TestNOW™ COVID-19 NAb kits with the RapiRead™ reader, the smallest device intended for such purpose, were able to detect neutralizing antibodies with sensitivity similar to CLIA methods in vaccinated and non-vaccinated subjects [56]. However, it has to be remarked that although this and other similar expedients can increase the sensitivity of these assays, additional equipment, preparation steps and a prolonged testing time are required. This makes difficult the use of these assays as POC tests, although the advantage that less amount of reagent is needed compared to standard centralized tests [57].

### 2.5. The role of antigens in LFIA

Antigen used for the lateral flow assays can include the S protein (S1 and S2), the N protein, the RBD or a combination of these antigens that are highly immunogenic and main targets for antibody response [17]. The choice of the targeted SARS-CoV-2 antigen in LFIA is fundamental. According to some authors, the accuracy of the test is strictly associated with the choice of the antigen and differences observed between assays can be partially explained by differences in the targeted antigens [16].

It is noteworthy that the kinetics of anti-N and anti-S antibodies are different, thus influencing the interpretation of the test [35]. As an example, antibodies against N proteins are longer-lived and more abundant than antibodies against other viral components, and seem more sensitive than the S protein antibody for detecting early infection, although this is not confirmed by all studies [58],[59]. In addition, N protein targeting assays, differently from S protein-based assays, can detect antibodies from SARS-CoV-2 infection, distinguishing it from vaccination-induced immunity [60],[61].

Unfortunately, as far as LIAFs are concerned, not all manufacturers disclose the antigen(s) used in their test, although this information is fundamental particularly to test during vaccination campaigns.

In a recent review, among 20 FDA-EU LFIAs for antibodies detection, half of the tests were based on S/S1 antigens alone or in combination with N. The remaining tests contained RBD alone or in combination with N and only 3 tests contained the N antigen alone [59]. For 3 LFIAs the antigen was not declared. All these assays showed a very high sensitivity, so that making a comparison between them is difficult [59].

### 2.6. Neutralizing antibodies

The protection induced by vaccines against SARS-CoV-2 has become in the last two years a topic of enormous interest requiring an in-depth knowledge on the immunological changes induced by SARS-CoV-2 and particularly on the role of neutralizing antibodies (NAbs). In fact, NAbs are a subset of the total polyclonal response to the virus whose relevance has emerged for their strong correlation with the immune protection induced by vaccines [62],[63]. The specific target of NAbs is the RBD located on the S1 domain of the S protein. The RBD is responsible for the high affinity binding of SARS-CoV-2 to the Angiotensin-Converting Enzyme-2 (ACE2) receptor, which is present on both human epithelial and endothelial cells [64].

Piccoli and coworkers in a landmark study analyzed the specificity and kinetics of neutralizing antibodies elicited by SARS-CoV-2 from a large number of infected subjects and found that the RBD is immunodominant in terms of total antibodies elicited, and is the target of 90% of the neutralizing activity found in the sera/plasma of most individuals evaluated [47]. Several studies now confirm that NAbs against RBD, blocking the interaction with ACE2 receptor, provide the most effective mechanism to neutralize the virus [65]–[67].

A review including 150 papers indicates that on average NAbs are detectable within 7–15 days following the disease onset, with levels increasing until 14–22 days, before reaching a plateau and then decreasing [66].

Kim and co-workers explored factors affecting titers and longevity of NAbs in post-COVID-19 patients. They found that, in all patients, titers decreased from 6 to 9 months after the infection, and that titers were higher in those with moderate-severe disease compared to those with mild disease [67]. Although the topic is still debated, some authors speculate that, as high titers of SARS-CoV-2 antibodies correlate with disease severity and mortality, it is possible that the overproduction of antibodies against SARS-CoV-2 induces disease progression via antibody-dependent enhancement, which is well documented for other viral infections [68],[69].

Khoury and co-workers, analyzing the relationship between in vitro neutralization level and the protection produced by either seven different vaccines or natural infection found that the neutralization level was highly predictive of immune protection [65]. This and other studies underline the relevance of the quantification of NAbs for a number of purposes and to find sensitive and specific assays to be used on large scale [70]–[72].

The quantification of NAbs in serum or plasma is obtained using specific neutralization assays including the plaque-reduction neutralization test and the micro-neutralization assay, considered the gold standard [73],[74]. These tests evaluate the effectiveness of plasma/serum from infected individuals to inhibit viral infection of target cells, thus measuring the antibody antiviral activity [74].

However, performing this assay is complex, requiring cell culture facilities, the use of the actual pathogen and Biosafety Level 3 laboratories, such that most of common laboratories are not properly equipped [74]. Alternatively, pseudovirus-based neutralization tests have been developed in which SARS-CoV-2 proteins are expressed by a surrogate virus [75]. Protein-based surrogate neutralization ELISA is another alternative assay [76].

In this context it has been proposed that lateral flow technology for qualitative detection of Nabs could be performed using the interaction between the S unit/RBD and the ACE2 receptor [29],[77]. However, although some possible technical solutions and alternative approaches have been proposed, the capacity of LFIA to detect NABs is so far unproven.

## 3. Currently available LFIA devices

The rapid spread of the pandemic rushed manufacturers to produce affordable assays such as LFIAs to be used as POC tests for antigens or antibodies detection. As mentioned, the FDA by the EUA procedure has given an emergency approval to a number of diagnostic tests and in cooperation with the Center for Disease Control (CDC) has provided alsoguidance on the development standards, safety and use of diagnostic technologies for COVID-19 [33],[35] Recommendations on the use of serological tests have also been published recently by the European Centre for Disease Prevention and Control [5].

As of February 16^th^, 2023 a total of 25 rapid antibody diagnostic tests remain granted through an EUA by the FDA [30]. Several CE-marked antibody assays are also available in the EU/EEA area [5],[78]. In addition, some other LFIAs have been approved by other regulatory institutions across countries [78]. A complete overview of available tests is provided by the FIND, an independent performance database which is continuously updated with serological tests and their performance characteristics, as evaluated by multiple partner institutions [79]. Noteworthy, the performance of commercialized rapid tests is variable and some of them have shown little reliability once on the market, such that the EUA has been revoked by the FDA for seven rapid tests that did not meet the clinical performance required to meet efficacy and risk/benefit standards [78].

In their review Ochola and coworkers show the percentage of lateral flow kits for serology that have been approved by different regulatory bodies across different countries in the world [80]. Filchakova and co-workers have recently published a list of the best performing strip immunoassays including LOD values and clinical sensitivity and specificity [81]. Interestingly, the sensitivity of FDA-approved assays was slightly higher compared to non-FDA-approved tests [37],[81]. Another inclusive list of rapid assays can be found from a recent article by Deshpande and co-workers [82].

## 4. Performance and reliability of LFIA in post-infection

To establish the accuracy of LFIAs, several performance parameters must be evaluated considering the intended use case. Generally, the product specifics include sensitivity, specificity, positive predictive value (PPV) and negative predictive values (NPV). In addition, it has to be considered, the inter and intra-operator reproducibility and analytical sensitivity (LOD) [80].

For detecting antibodies from prior SARS-CoV-2 infection, the WHO considers “acceptable” a POC test with ≥90% clinical sensitivity and ≥95% clinical specificity and “desirable” test with ≥97% sensitivity and ≥95% specificity [37]. Within the first 3–4 months after the initiation of the pandemic the few commercially available LFIAs were used to detect antibodies, mainly in patients with acute infection by SARS-CoV-2 [15],[38],[83]. Throughout this period, these tests showed a variable sensitivity and specificity for IgG and particularly for IgM detection [16],[84]. Indeed, the main problem in establishing the performance of these as other serological tests were the lack of guidelines for comparing methods. Although virus neutralization is considered the gold standard for antibody detection, in the vast majority of cases the comparison was made between LFIAs and qualitative methods such as ELISA or CLIA [84].

It must be remarked that the accuracy of a POC is variable not only due to differences in manufacturers' assays or dosing methods, but also due to the time frame of the performance [40]. If IgG levels are to be detected, these will reach a maximum titer and plateau after 2/3 weeks after symptoms onset; therefore, over this period the best diagnostic sensitivity will offer more relevant information on long-term immunity [40]. In addition, differences in the population studied must be considered, as these can include symptomatic and asymptomatic infected patients, convalescents and re-infected [57]. Other factors include the specific environment or individual factors such as age and comorbidities that can be involved in the immune response [57],[85]. Taking together these considerations it is not surprising that data on the accuracy of LFIAs may be contrasting.

As mentioned, in the first phase of the pandemic LFIAs for antibodies detection were used with diagnostic purpose as auxiliary tests to RT-PCR [15],[37],[38],[86]. As Italy was the first European country to be dramatically affected by the pandemic, our group performed one of the earliest studies, as soon as the first LFIA kits for IgG and IgM became available from China. In infected patients overall the seroconversion rate for IgG/ IgM was 82% after a median time of 18 days and the sensitivity and specificity were 83% and 93%, respectively, compared to RT-PCR [15]. A larger analysis showed a good performance of eight FDA-approved POCs meeting the WHO criteria with a mean clinical sensitivity of 98.3% (CI 83–100) and a specificity of 98.2% (CI 87–100) [37]. The clinical sensitivity and specificity of these tests were significantly higher compared to other tests from the FIND database [31]. Although a large number of studies have successively investigated LFIAs during the acute illness, it is accepted that serological tests should not be performed before 2 weeks from the onset of the infection, independently from the test used and this has been confirmed by the European Centre for Disease Prevention and Control [5]. In addition, it has to mentioned that although the molecular test is the gold standard for the diagnosis of SARS-CoV-2 infection, thus used to validate the accuracy of LFIA, this test may misdiagnosis the diseases, giving both false positive or negative results [87].

In the acute phase, some authors have tried to correlate antibody detection by LFIA and clinical or imaging findings. In one study on 320 patients with confirmed COVID-19, the rate of chest computed tomography (CT) findings was significantly higher in patients with antibody positivity than in those with antibody negativity [88]. This was confirmed by another study showing that increasing severity of lung involvement is associated with high and persistent IgG antibody titers [89]. It is noteworthy that studies correlating more severe disease to more vigorous antibody response in COVID-19, are performed using laboratory tests, and not LFIAs, to assess the antibody titer [90]. Recently Jurenka and co-workers using a LFIA kit assessed anti-S SARS-CoV-2 antibodies in hospitalized patients and found that the absence of IgM and IgG at the time of admission predicted in-hospital mortality [91]. However, results from this study are in sharp contrast with previous findings [68].

### 4.1. Comparison between LFIA and laboratory tests

A large number of studies evaluated the accuracy of LFIA for antibody detection compared to ELISA, CLIA or RT-PCR and some meta-analyses and reviews have summarized results of these studies [11],[84],[86],[91]–[95]. Deeks and colleagues in an early Cochrane review, including sparse data collected in various time ranges from the disease onset, found inconsistencies between the available assays, however, POCs had higher pooled sensitivity than ELISA for IgG and IgG-IgM [86]. Successively, other meta-analyses have confirmed the discrepancies between the tests, reporting a low pooled sensitivity and a high specificity of LFIA for both IgG and IgM [26],[84], [92]–[95]. Vengesai and co-workers evaluated the accuracy of different serological tests for IgG (17 studies) IgM (16 studies) and IgG-IgM (27 studies) and found that overall the most sensitive test for IgG was CLIA, whereas ELISA had the highest diagnostic accuracy for combined IgG-IgM [91]. The largest meta-analysis performed on LFIA by Gracienta et al. included 33 studies and showed that the combined detection of IgM and IgG had the highest sensitivity and NPV of 93%, whereas the single IgG detection method had the highest specificity and PPV of 96.68% [95]. The Cochrane review on antibody testing published in 2020 by Deeks et al. has been updated in 2022 and confirms that CLIAs are the most sensitive and specific tests for detecting IgG compared to both ELISAs and LFIAs [96]. Taken together data from available meta-analyses indicate: 1) lower sensitivity of LFIA compared to laboratory tests although a high specificity; 2) lower sensitivity for IgM compared to IgG for every serological methodology; 3) greater accuracy of the detection of combined IgG/IgM compared to the detection of a single antibody [26], [95],[97],[98].

Indeed, although presenting a large number of studies pooling a high number of samples, these meta-analyses present many limits including mainly: 1) heterogeneous methods and study designs; 2) most of the studies are case control rather than cross-sectional; 3) different study populations; 3) different types of kits, detecting antibodies against different virus proteins; 4) different time of sample collection, in some cases being also <14 days. Given the enormous number of kits quickly put on the market, the inclusion of tests that are insufficiently validated is another putative reason for inconsistent results [26]. Of course, one limitation of our conclusion is that this is a narrative review and we did not systematically revised the articles we cited and possibly we have omitted some work.

Soon after the implementation of rapid tests on a large scale, a number of problems have emerged. One of the reasons for testing individuals and populations is to assess the level of protection against SARS-CoV-2 and LFIAs are mainly qualitative tests, with limits of detections varying between different brands [82]. Therefore, a positive rapid test does not necessary mean that protection is present. In addition, the antibody titer necessary to achieve protection has been debated, although some recent large trials are providing evidence on the immune biomarkers that correlate with protection and on threshold titers to be achieved to obtain sustained protection [99],[100]. It has also to be mentioned that even with centralized laboratory tests it has been difficult to compare cut-off values, since antibody titers are not harmonized according to the WHO International Standard for anti-SARS-CoV-2 immunoglobulin [101]. To meet these needs, research is ongoing to transform quantitative LFIAs to semi-quantitative tests, in which the intensity of the visual band can be correlated with the level of antibodies [102],[103]. Also, studies correlating LFIAs results with the rate of protection would be useful. Interestingly, one study showed that positive IgG results on four different LFIAs were associated to a lower rate of subsequent SARS-CoV-2 infection, and these results were similar to those obtained with laboratory immunoassays [104]. However, this topic needs further investigation.

New LFIA kits have been produced and further studies on serology have been published in this last year. Desphande and co-workers in an extensive revision of 22 LFIAs found a good sensitivity and specificity for many of these tests, with the PrimaLAb, detecting the S1 protein, showing 100% sensitivity [82]. Moreover, Choi and co-workers recently found, in 988 serum samples, a strong positive correlation between quantitative results obtained by four POC assays for SARS-CoV-2 antibodies detection and results obtained by a surrogate virus neutralization [75]. In this study the sensitivity of the rapid assays in symptomatic patients increased from 56% in the first week to 92% after 3 weeks and then decreased to 80% after >29days [105]. Pan and co-workers pooled serological data from 35775 participants from all the continents finding a good predictive value for SARS-CoV-2 infection, however data from POCs were not specifically analyzed [106]. Key considerations for evaluation of studies on LFIA have been formulated by Ochola and co-workers [80]. (Table 2).

**Table 2.** Aspects to be considered when evaluating or designing studies on LFIA for the detection of antibodies against SARS-CoV-2.

Aspects
Target population, sampling scheme and study case definition
Prevalence in the target population Performance characteristics (sensitivity, specificity, positive and negative predictive value) Specificity controls (cross-reactivity against other seasonal coronavirus) Reference standard Limits of detections Target antigen Isotype of interest Variants and mutation of the antigen Vaccination status Use cases Financial effectiveness Impact on decision clinical making and utility for the health system

Adapted from Ochola et al. ref n. 80

### 4.2. Relevance of timing

**Figure 2.**
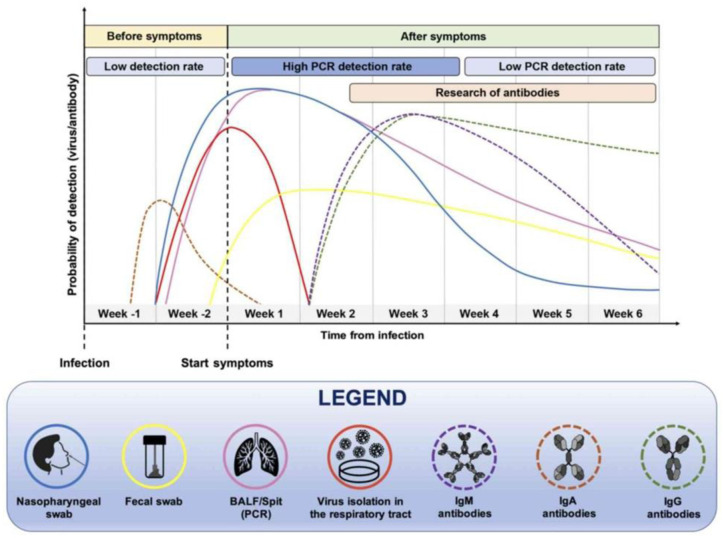
Timing for effective anti-SARS-CoV-2 IgA, IgM and IgG detection and the 343 relationship with the detection of SARS-CoV-2 RNA in different samples (from Falzone L et al. ref n. 144).

The time point of sample collection is crucial when establishing the accuracy of serological tests (Figure 2). A meta-analysis pooling 11516 samples clearly shows the increase in sensitivity of serological tests from day 7 to day 21 from disease onset [49]. Similar results have been shown specifically for LFIAs [82],[84]. Desphande and co-workers found that for most LFIAs the sensitivity increased considerably after 2 weeks from the disease onset, giving better results with IgG detection rather than IgM. The sensitivity of the assays remained high up to 25 days [82]. In one recent systematic review it was shown that the sensitivity of LFIA after 21 days approached the sensitivity of the ELISA method [94]. Although the permanence of IgG after SARS-CoV-2 infection can be long-lasting, up to one year, the problem is that little is known on the reliability of LFIAs in detecting antibodies over the time (>30 days from the infection) and this would be important when conducting long-term surveillance among populations [104],[108]. In one study a POC test for IgG exhibited a high sensitivity comparable to ELISA and CLIA up to 60 days from the infection [109].

Our group has compared the accuracy of LFIA versus ELISA and CLIA in detecting IgG over the time in patients previously infected with SARS-CoV-2. We found that the sensitivity of LFIA at 3–4 and 6–7 months after the infection was 96% and 95%, respectively, thus remaining within desirable criteria for POC tests [110]. In addition, after 6 months we found a strong correlation between results obtained by LFIA and those obtained by ELISA and CLIA (Table 3). These data are in accordance with another study, where IgG could be detected by LFIA with a sensitivity of 97% up to 10 months after the infection [111].

**Table 3.** Accuracy and predictive values of LFIA for SARS-CoV-2 IgG in 229 previous infected subjects from 3 to 6 months after the infection (from Spicuzza L et al. 2022).

	Sensitivity (95% CI)	Specificity (95% CI)	PPV	NPV (95% CI)	Accuracy (95% CI)
Overall	95.5 (91.7–97.9)	100 (78.1–100)	100	63.0 (47.3–76.3)	95.9 (92.3–96.1)
3–4 months	96.4 (88.7–99.2)	100 (54.0–100)	100	67.8 (40.9–86.4)	96.6 (90.5–99.3)
6–7 months	95.0 (89.4–98.1)	100 (66.3–100)	100	60.0 (40.8–76.6)	93.3 (90.1–98.2)

Indeed, the optimization of a standardized approach for the correct timing of serology tests remains challenging, but extremely important to consider LFIA a useful tool for population surveillance [112].

## 5. LFIA for seroprevalence studies

Seroprevalence studies estimate the rate of exposure to SARS-CoV-2 in a target population, taking into account that many patients remain asymptomatic, thus giving an indication of under-ascertainment of reported infections. Therefore, serology is fundamental for population-based epidemiological surveillance that can help public health in policy making, to address the extent of SARS-CoV-2 spread in the community and the effectiveness of control strategies [112], [20]. Qualitative antibody tests are useful from a population-wide, rather than individual perspective [5]. LFIA is superior to ELISA for large-scale evaluation in aspects of detection cost, detection time and operation process [80],[113]. Generally, screening includes high risk groups such as health care and public workers. In an early prospective multicenter cohort study Pallet and co-workers compared the usefulness of POCs and laboratory serology assays for late case identification among high risk health workers in UK [84]. A good positive predictive value was observed with both LFIA and ELISA [114].

According to the European Centre for Disease Prevention and Control the individual possibility of a positive antibody test is dependent on the individual exposure and likelihood of infection over the time [5]. In fact, one of the early studies has found that the proportion of false positives can differ drastically from 3% to 88% in individuals from areas with different prevalence of the infection, while the false negative rate is typically below 10% [115]. However, this problem has been overcome by the fact that over the years comparable epidemiological situations have been observed in western countries, although data are uncertain for some low-income countries.

Currently, as a large part of the global population is vaccinated, seroprevalence data in non-vaccinated are investigated in small population samples particularly in sub-Saharan Africa [80]. For this purpose, it is important to note that LFIAs targeting the N protein can detect antibodies from SARS-CoV-2 natural infection, whereas LFIAs targeting the S protein are not able to distinguish natural infection from vaccination [60].

## 6. LFIA and response to vaccination

Following the widespread of vaccination against SARS-CoV-2 across the continents, testing to determine the level and the duration of the protection, both in populations and in individuals, has been considered fundamental. In addition, as the vaccine campaign was conducted while the pandemic was ongoing and often in a peak phase, serology helped to address policy of vaccine administration to previously infected subjects. In fact, the presence of antibodies against the N protein indicated a past infection and in naturally immunized individuals vaccination could be delayed for some time. Moreover, those with past infection who received a single dose of vaccine developed a titer of NAbs nearly three times higher compared to those never exposed, thus making unnecessary a booster dose [57],[116].

Theoretically, LFIA should be superior to laboratory tests for large-scale evaluation of vaccine effectiveness due to lower costs and detection time [113]. However, although those LFIAs using the protein pair RBD/ACE2 can perform accurately for certain applications, in practice a number of problems have risen up in the use of rapid tests in post-vaccination [89],[90]. No doubt that an association exists between the efficacy of the vaccine and neutralizing antibody titers, but comparison of immune responses provided by different vaccines is challenging and different approaches have been used by vaccines developers [117],[118].

The most commonly used vaccines by Moderna and Pfizer-BioNTech induce immunization by introducing mRNA that tricks the body to produce endogenously viral spike proteins [119]. The Moderna vaccine targets the production of antibodies towards the receptor RBD and this can cause interference in tests including the RBD colloidal gold conjugates. The Pfizer-BioNTech vaccine produces a full length S protein and other S fragments including the RBD, with a theoretical possibility to cause complement binding for the S protein antibody [119],[121].

Recently Mulder and co-workers analyzed nine different LFIAs, with target N, S or both proteins on samples from post-infected and post-vaccinated (mRNA and inactivated virus vaccines) individuals [102]. A great variability in the sensitivity of the tests has been reported. The performance of the spike protein-containing assays was better than N-protein based tests in identifying post-vaccination immunity, although these tests were not able to distinguish post-vaccination from post-infection. Therefore, the choice of the most appropriate test, based on the target antigen is mandatory. Some studies however, have failed to find a desirable sensitivity of LFIA in the response to vaccination, particularly if intended for individual testing [119].

One problem is that, after administration of the most common mRNA vaccines, antibody titers may vary among subjects as well as over the time, so that some subjects can show low if any level of antibodies after vaccine administration [122]. In these cases, it is unclear whether a rapid antibody test will have the sensitivity to yield a positive result [119]. Furthermore, it is also possible that vaccinated individuals may develop NAbs binding to non-RBD domains making difficult their detection [117].

However, there are some studies on this field that seem encouraging. Cann and co-workers recently published that the Fortress LFIA test, detecting anti-spike IgG, performed well compared to the widely used Abbott Architect SARS-CoV-2 IgG in a group of vaccinated individuals [123]. Another study, using a LFIA to assess the presence of RBD-ACE2 NAbs in whole blood (COVID-19 NAb-testTM) in a cohort of vaccinated subjects reported a specificity of 100% and a sensitivity of 96% compared with microneutralisation [124]. Some groups have attempted a quantitative detection using lateral flow technology. One group performed quantitative detection of S-RBD in fingerstick whole blood with the FinecareTM Test combined with the FinecareTM reader and found a good performance compared to surrogate virus-neutralizing assay and to three highly performing automated immunoassays, both in post-infection and post-vaccination [125]. Another novel microfluidic cartridge-based device (ViroTrack Sero COVID-19 Total Ab) was used for quantitative detection of total antibodies against SARS-CoV-2 trimeric spike protein showing a good accuracy compared to standard CLIA in subjectsinjected with the Pfizer-BioNTech vaccine [126]. Interestingly, Tong and co-workers integrated a polydopamine nanoparticles-based LFIA and a smartphone-based reader to test NAbs in serum, using an artificial intelligence to quantitatively analyze results. They showed that this system was more accurate than AuNP based LFIAs, to assess NAbs in samples from vaccinated individuals [113].

A very recent study assessed the sensitivity of eight LFIAs in post-vaccinated individuals and found that the sensitivity increased from dose one to dose two in six out of eight LFIAs [127]. Three tests detecting anti-spike antibodies achieved 100% sensitivity at dose two, compared with a chemiluminescent microparticle immunoassay [127]. Another recent study showed that a LFIA (Humasis®, Anyang, South Korea) was able to detect anti-spike antibodies in plasma samples of vaccinated subjects with an accuracy comparable to ELISA, and the strength of label bands correlated with antibody concentrations, although effective protection against infection was not assessed [128].

An effort has been done to establish a putative role for LFIA in determining the longevity of vaccine-induced protection. One group followed-up the response to BNT162b2 by three different LFIAs detecting N protein, S1 subunit and RBD NAbs in the sera of 107 health care workers up to 8 months from vaccination [129]. After three weeks, the RBD-based LFIA showed a sensitivity between 98% and 100%, but after 8 months this sensitivity significantly dropped compared to laboratory quantitative tests. Conversely, Wang and colleagues found that the lateral flow NeutraXpressTM was accurate, compared to standard laboratory methods, to monitor the wane in mRNA vaccine-induced NAbs after 3–6 months from vaccination [130]. More recently, a study performed on 107220 individuals up to 30 weeks after primary vaccination, showed a good correlation between results obtained with LFIAs and neutralizing antibody titres [131].

Indeed, the research on LFIA for post-vaccination monitoring is quickly growing, for the undisputed previously described advantages of these tests. However, it has to be mentioned that some authors agree that currently antibody detection by LFIA, although useful for some intended purposes, is not ideal to evaluate vaccine-induced immune response and the use in post-vaccination monitoring is not yet recommended [29],[77],[118].

## 7. Impact of new variants of SARS-CoV-2

After the spread of the original strain of SARS-CoV-2 from Wuhan in 2019 the emergence of new variants has heavily challenged the control of the pandemic. Five of these have been of major concern for the rapid spreading including Alpha (B.1.1.7), Beta (B.1.351), Gamma (P.1), Delta (B.1.617.2) and the Omicron (B.1.1.529) variant being dominant worldwide from the beginning of 2022 [132]. These new variants, each to a different extent, are able to enhance viral replication and transmission, raise the risk of reinfection and blunt the potency of neutralizing antibodies induced by previous infection or vaccination [132]. Most mutations occur in the spike proteins and those affecting RBD are of course particularly important. For diagnostic purposes it is clear that if the mutation occurs in that part of the viral genome that produces proteins detected by antigen tests or responsible for antibodies production, this will affect the performance of the diagnostic assays.

Currently, the highly transmissible omicron variant has expanded into more than one hundred sublineages with a high number of spike protein mutations, allowing the virus to escape from the immunity produced by vaccination against the original strain [133],[134]. One important mutation is the R346X, situated in the RBD with enhanced capacity to escape neutralizing antibodies [135]. Very recently the two omicron variants BQ.1.1 and XBB.1.5, the second also known as the “Kraken” subvariant, have shown the ability to escape treatment with monoclonal antibodies against earlier omicron variants [136].

New vaccines against omicron BA4-5 or bivalent vaccines against original strain/omicron BA4-5 have been produced and already implemented in most countries [137]. In addition, trimeric spike-based vaccines are under evaluation, eliciting cross-neutralization against different variants including Beta, Delta and Omicron [138]. Of course, the highly mutagenicity of the S protein in the omicron variant can impact the performance of S-based LFIAs or other serological assays, whereas the mutation of the N unit is generally rarer [139]. One study investigating serum NAbs against omicron in vaccinated or natural infected subjects found that, compared to virus neutralization tests, commercial available CLIAs had a sensitivity and specificity of 89% and the best results were obtained with RBD-IgG tests [140]. Studies have now clearly shown a low sensitivity of common antibody assays, containing S or RBD antigens, in detecting antibody in patients infected with omicron BA.1 or BA.2, compared to the ancestral variant [141],[142]. These studies s most commonly considered ELISAs [141],[142]. New laboratory and POC tests to detect antibody response to VOCs are currently under evaluation. Recently, a POC antibody microarray, termed CoVariant-SPOT, has shown the ability to distinguish between the two VOCs Delta and Omicron [143].

## 8. Conclusion

After the onset of the COVID-19 pandemic serological tests have been included among strategies to recognize the actual spread of the pandemic, still ongoing with alternating phases due to the emergence of new variants. In this context, the lateral flow technology for antibody detection has become one of the most convenient and widely used alternatives to laboratory tests. Numerous studies have been published on the accuracy of LFIA for antibody detection and some meta-analyses have concluded that these assays exhibit a good performance, however, although a high specificity, the sensitivity is lower when compared with ELISA and CLIA, so that LFIA should be used for large seroprevalence studies rather than for individual testing. Remarkably, some products, particularly among those approved by the FDA, have emerged showing a very good accuracy. Thus, whatever is the intend use, the choice of the test is fundamental.

Furthermore, a great amount of work has been undertaken to improve the performance of these tests, mainly improving the recognition and signal generation technologies, readout and signal transduction technologies and the design of the cartridges. With the implementation of vaccination campaigns, theoretically the POC tests should be the ideal candidate to assess the presence and the durability of vaccine-induced protection on large scale, being easy and rapid to perform. Studies are ongoing, but sound evidence on this potential use is still lacking, particularly after the unending emergence of virus variants escaping acquired immunity. In particular, the emergence of several antigenic changes in the Omicron variants causes a reduced immunoreactivity in the currently available S- and RBD-based antibody assays, impairing their diagnostic accuracy. However, the current trend toward rapid biomarker analysis seems unstoppable, and although LFIA is still far to be the “perfect” test, indeed it presents unquestionable advantages over more sophisticated tests, so that further research is granted.
